# Supplementary material for: A Systematic Scoping Review of Pre-School Self-Regulation Interventions from a Self-Determination Theory Perspective
Source: Int J Environ Res Public Health. 2022 Feb 21;19(4):2454. doi: 10.3390/ijerph19042454 (PMC8878745; doi:10.3390/ijerph19042454)
Supplement: Supplementary file 1 [file ijerph-19-02454-s001.zip › ijerph-1540500-supplementary/ijerph-1540500-supplementary Table S1.pdf]

Table S1. Data Extraction for Final Study Selection.

Author	Study Context	N	Age (*M*, months)	Geographical Location	SDT	Approach	Duration	Dose	Interventionist Training	Measure	Significant	Effect Size	Comparative Effect Size
Berti & Cigala (2018)	Kindergarten	21	65.6	Italy	None	Mindfulness	6 weeks	15 sessions/wk	No — Trained instructor	HTKS	No	−0.07	No effect
Blair et al. (2018)	Preschool	759	69.0	United States	CRA	Curriculum	9 months	Daily	2-year *Tools* professional development	Teacher rates classroom behaviour scale (SR composite)	Yes	0.18	Small
Diamond et al. (2019)	Preschool	352	-	Canada	CRA	Curriculum	9 months	Daily	3-day workshop prior to intervention, 4 1-day workshops throughout the academic year	Teacher reports of attention regulation and self-control. 'Ability to get back to work after a break' and 'ability to work independently without supervision'	Yes	Ability to get back to work after a break = 1.29 Ability to work independently without supervision = 1.22	Large
Duncan et al. (2018)	Preschool	567	66.2	United States	AC	Child Activities	3 weeks	20–30 min daily	3-hour workshop	HTKS	Yes	0.32 *	Small
Flook et al. (2015)	School	68	56.1	United States	C	Mindfulness	12 weeks	40–60 min per week	No- Trained instructor	TSC, sharing task, DoG.	Yes (not for DoG)	TSC = 0.32, sharing task = 0.62, DoG = 0.23	Small – moderate
Howard et al. (2020)	Preschool	473	53.3	Australia	RC	Child Activities	6 months	6 activities/wk	9 online professional development videos	HTKS, PRSIST, CSBQ by educators	No	HTKS 0.003 [a], PRSIST 0.012 [a], CSBQ 0.005 [a]	Small
Jackman et al. (2019)	Preschool	262	44.0	North America	R	Mindfulness	9 months	Daily	Teachers trained via 5-day mindfulness course, and parents 3 x 2hr mindfulness sessions	HTKS	Yes	0.43	Small

Jelley et al. (2016)	HLE & Preschool	150	-	United Kingdom	RA	Child Activities	18 weeks	19% accessed daily	No	CSBQ by parents	Cognitive subscale only	Cognitive subscale 0.47<br><br>Behavioural subscale 0.08<br><br>Emotional subscale 0.06	Small
McClelland et al. (2019)	Preschool	157	51.0	United States	CRA	Child Activities	8 weeks	15–20 min twice a week	Teachers received half a day training	HTKS	No	RLPL 0.25, RLPL+ 0.32, together 0.31	Small
Meuwissen & Carlson (2019)	Lab	128	39.5	United States	R	Parent behaviour manipulation	1 lab session	1 video modelling behaviour	Parents watched video matched to gender and condition, modelling behaviour	Coding frame	No	Not available — On average children became less well regulated.	No effect.
Poehlmann-Tynan et al. (2016)	Preschool	29	47.0	United States	C	Mindfulness	12 weeks	40–60 min/wk	No — trained instructors	HTKS	Yes	0.26[a]	Large
Razza et al. (2015)	Preschool	29	51.1	United States	R	Mindfulness	25 weeks	Daily	200h in YogaKids certification	Toy Wrap, Toy Wait, HTKS	Toy Wrap only	Toy Wrap 0.22 [a], Toy Wait 0.13 [a]<br><br>HTKS 0.14 [a]	Moderate
Robinson et al. (2016)	School	113	51.9	United States	AC	Physical	5 weeks	3 × 40min/wk	No — experienced instructors	DoG	Yes	0.97	Large
Schmitt et al. (2015)	Preschool	276	51.7	United States	AC	Child Activities	8 weeks	20–30 min twice a week	One training workshop	CBRS, HTKS	HTKS only	HTKS 0.32<br><br>CBRS not published	Small
Sezgin & Demiriz (2019)	Preschool	54	56.7	Turkey	AC	Child Activities	8 weeks	3 days/wk	Not published — interventionist unknown	CBRS, HTKS	HTKS only	2.8<br><br>CBRS not published	Large
Shiu et al. (2020)	Kindergarten	94	67.0	Taiwan	AC	Child Activities	12 weeks	120 min, 3 days/wk	No — led by researcher	HTKS	Yes	0.4	Small

Solomon et al. (2018)	Childcare centres	195	45.5	Canada	CRA	Curriculum	15 months	Daily	Manual followed, including workshops and in-class coaching	HTKS	No	Not published	Small*
Taylor & Butts-Wilmsmeyer (2020)	Preschool	385	55.8	Canada	R	Nature	15 weeks	Low frequency 60 min/wk, high frequency 30–60 min/day	No	HTKS, CBRS	Only for group × gender	Study 1: HTKS 0.05 [a], CBRS 0.03 [a]<br><br>Study 2: HTKS 0.01 [a], CBRS 0.0001 [a]<br><br>Only effective for boys: HTKS 0.04 [a], CBRS 0.03 [a]	No effect - small
Tominey & McClelland (2011)	Preschool	65	54.6	United States	AC	Child Activities	8 weeks	30–60 min, twice a week	No — led by researcher	HTKS	No	0.10	Small
		4177	54.5										

Note. RLPL (Red Light Purple Light); RLPL+ (standard RLPL with additional literacy intervention called Bridge to Kindergarten (B2K); CHAMP (Children's Health Activity Motor Program); KC (Kindness Curriculum); Tools (Tools of the Mind); OM (Open Mind); PRSIST (Preschool Situational Self-Regulation Toolkit), HLE (Home Learning Environment). HTKS (Head Toes Knees Shoulders), TSC (Teacher-rated Social Competence); DoG (Delay of Gratification), PRSIST (Preschool Situational Self-Regulation measure), CSBQ (Child Self-Regulation & Behaviour Questionnaire), CBRS (Child Behaviour Rating Scale). Effect sizes are presented as Cohen's d unless outlined below. [a] Partial eta-squared. * Effect size was extracted, imperfectly, from other aspects in the paper.
